# Occurrence of triatomines (Hemiptera, Reduviidae) and their natural infection by *Trypanosoma cruzi* (Chagas, 1909) in Boca do Moa community, Cruzeiro do Sul, Acre, Brazil

**DOI:** 10.1590/0037-8682-0590-2020

**Published:** 2021-03-08

**Authors:** Adila Costa de Jesus, Fernanda Portela Madeira, Madson Huilber da Silva Moraes, Adson Araújo de Morais, Jader de Oliveira, João Aristeu da Rosa, Luís Marcelo Aranha Camargo, Dionatas Ulises de Oliveira Meneguetti, Paulo Sérgio Berrnarde

**Affiliations:** 1 Universidade Federal do Acre, Programa de Pós-Graduação Stricto Sensu em Ciências da Saúde na Amazônia Ocidental, Rio Branco, AC, Brasil.; 2 Universidade Federal do Acre, Campus Floresta, Centro Multidisciplinar, Laboratório de Herpetologia, Cruzeiro do Sul, AC, Brasil.; 3 Hospital Sírio-Libanês, Sociedade Beneficente de Senhoras, Programa de Pós-Graduação Lato Sensu em Especialização em vigilância em Saúde, São Paulo, SP, Brasil.; 4 Universidade Estadual Paulista Júlio de Mesquita Filho, Faculdade de Ciências Farmacêuticas, Departamento de Ciências Biológicas, Araraquara, SP, Brasil.; 5 Universidade Estadual Paulista Júlio de Mesquita Filho, Programa de Pós-Graduação Stricto Sensu em Biociências e Biotecnologia, Araraquara, SP, Brasil.; 6 Universidade de São Paulo, Instituto de Ciências Biomédicas 5, Monte Negro, RO, Brasil.; 7 Centro Universitário São Lucas/Afya, Departamento de Medicina, Porto Velho, RO, Brasil.; 8 Centro de Pesquisa em Medicina Tropical de Rondônia, Porto Velho, RO, Brasil.; 9 Instituto Nacional de Epidemiologia da Amazônia Ocidental, Porto Velho, RO, Brasil.; 10 Universidade Federal do Acre, Colégio de Aplicação, Rio Branco, AC, Brasil.

**Keywords:** Chagas disease, Trypanosomatids, Western Amazon

## Abstract

**INTRODUCTION:**

Triatomines are insect vectors of *Trypanosoma cruzi*, the etiological agent of Chagas disease.

**METHODS:**

Triatomines were collected from households and by dissecting palm trees in the peri-urban areas of Cruzeiro do Sul (Acre); they were identified using a specific key and via genital analyses. Trypanosomatid infection was determined through microscopy and polymerase chain reaction.

**RESULTS:**

In total, 116 triatomines of the species *Eratyrus mucronatus*, *Rhodnius pictipes*, *R. stali,* and *R. montenegrensis* were collected, of which 13.8% were positive for *T. cruzi*.

**CONCLUSIONS:**

Four species of triatomines presented an infection rate above 13% in the Boca do Moa community.

Chagas disease, transmitted by hematophagous insects of the family Reduviidae belonging to the subfamily Triatominae^1^, is caused by the etiologic agent *Trypanosoma cruzi.*

Of the more than 30 species of hematophagous insects present in the Amazon^2^, 11 are present in the state of Acre, 8 of which were recorded from 2013 to 2019^1^ ^,^ ^3^. However, most of these records were reported in the Vale do Acre mesoregion, located in the east of the state. There are very few studies in Vale do Juruá, located in the west of the state.

 Therefore, the objective of this study was to analyze the occurrence of triatomines and their natural infection by *T*. *cruzi* in the Boca do Moa community, a peri-urban region in the municipality of Cruzeiro do Sul.

The Boca do Moa community (7° 39' 22, 7"S and 72° 40' 48, 5"O) is located in the mesoregion of Vale do Juruá, approximately 3 km from the center of Cruzeiro do Sul, Acre (Figure 1), which is 648 km from the capital, Rio Branco. The community has a total area of 87,792 km² and an altitude of 193 m, and has 43 households where approximately 200 people reside.

**FIGURE 1:**
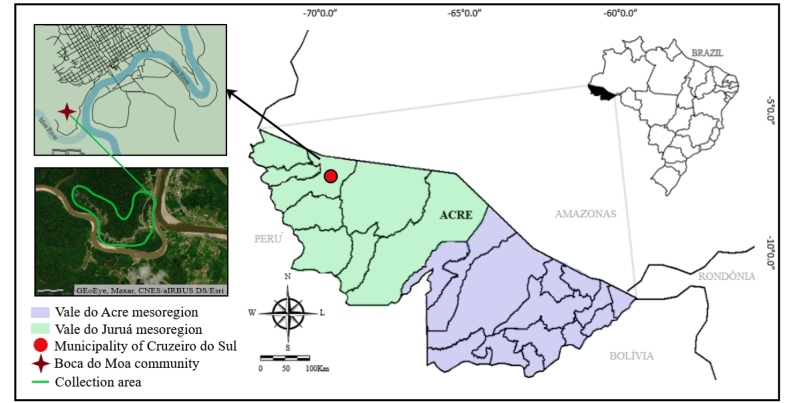
Geographic location of Boca do Moa Community in the municipality of Cruzeiro do Sul, Acre, Brazil.

Triatomines were captured in the period of August 2017 to July 2018 through third-party collection and dissection of palm trees. Third-party collection occurred through a passive search, where 43 resident family units were provided with collector flacks to capture specimens found in their homes. The active search through the dissection of palm trees was performed in the mornings and involved the cutting of Jaci palm tree (*Attalea butyracea)* (*Mutis* ex L.f.) bracts using chainsaws. Immediately after cutting, the bracts were individually and gradually removed, and triatomines were collected manually.

The palm trees were selected for convenience owing to the presence of large crowns and being located between an open environment and a forest fragment. Previous studies by other researchers in locations with similar environmental characteristics as in this study area have shown great success in collection conducted in open areas^4^ located near households^5^.

All captured specimens were kept alive in plastic containers and sent in thermal boxes at room temperature for identification. Identification was done based on external morphological characters, using the identification keys by Lent and Wygodzinsky^6^, Rosa et al.^7^, and Galvão^2^ as parameters.

Then, the identity of the adult specimens (both male and female) of the genera *Rhodnius* was confirmed through characteristics of their internal genitalia as described by Rosa et al.^7^and Rosa et al.^8^. The specimens found to be in the growth stage (also known as nymph) were raised in captivity until they reached adulthood for identification.

Triatomine infection was analyzed using optical microscopy. The digestive content of the insect was diluted in 0.9% saline solution on slides and observed under 1000× magnification, both fresh and stained with Panótico Rápido® (triarylmethane 0.1%, xanthene 0.1%, and thiazine 0.1%). When positive, molecular analysis was performed according to a method reported by Fernandes et al.^9^. This method amplifies a portion of the non-transcribed spacer of the mini-exon gene that is different between *T. cruzi* and *T. rangeli* species and between *T. cruzi* strains. The generated fragments vary in length between 100 and 250 base pairs. The oligonucleotide sequences used were TC1: (5-ACACTTTCTGGCGCTGATCG-3); TC2: 250 bp, (5-TTGCTCGCACACTCGGCTGCAT-3); Z3: 150 bp, (5-CCGCGCACAACCCCTATAAAATG-3); TR: 100 bp, (5-CCTATTGTGCCATCTTCG-3) and EXON: (5-TACCAATAGTACACACAACTG-3′)^9^.

The reaction mixture consisted of 100 pmol of each primer and 150 μM deoxynucleotide triphosphates in a buffer consisting of 10 mM Tris-HCl (pH 8.3), 1.5 mM MgCl_2_, 25 mM KCl, 0.1 mg/ml bovine serum albumin, 2.5 U TaqDNA polymerase, and approximately 10 ng of the genomic DNA sample, totaling a final volume of 50 μL with Type 1 water^9^.

Thermal cycling conditions during each step were as follows: an initial step of 5 min at 95°C; 34 cycles of 30 s at 94°C, 30 s at 55°C, and 30 s at 72°C; and a final extension of 10 min at 72°C. The following reference strains were used as controls in each reaction: TC1 X10 Clone 1, TC2 strain Y, Z3 Esmeraldo Clone 1, and *T. rangeli* R1625. The amplified products were electrophoresed on a 2% agarose gel at 100 V for 1 h. After electrophoresis, the DNA was stained with ethidium bromide and visualized under ultraviolet light. A molecular marker of 50 base pairs was used as a size control for the amplified fragments^9^.

A total of 116 triatomines at all stages of development were collected, with the genus *Rhodnius* being the most representative (98.27%).

The species collected were *Rhodnius pictipes* Stål, 1872; *R. stali* Lent, Jurberg & Galvão, 1993; *R. montenegrensis* Rosa et al., 2012; and *Eratyrus mucronatus* Stål, 1859. The results also included *Rhodnius* sp. 1 (standard *R. montenegrensis/R. robustus* Larrousse, 1927) and *Rhodnius* sp. 2 (standard *R. pictipes*/*R. stali*), which were the specimens of the genus *Rhodnius* that did not reach the adult stage and thus could not be identified (Table 1).

**TABLE 1:** Triatomine species collected from palm trees and households and their *T. cruzi* infection.

Species	Growth stage	N.	N.	Positive	
		**(*A. butyracea*)**	(Residences)	**(*T. cruzi*)**	
				N	%
*Rhodnius montenegrensis*	Ni1	-	-	-	-
	Ni2	-	-	-	-
	Ni3	-	-	-	-
	Ni4	2	-	-	-
	Ni5	4	-	3	75
	A	12	6	5	27,8
	T	**24**		8	33,3
*Rhodnius pictipes*	Ni1	-	-	-	-
	Ni2	-	-	-	-
	Ni3	-	-	-	-
	Ni4	1	-	1	100
	Ni5	-	-	-	-
	A	-	-	-	-
	T	**1**		1	100
*Rhodnius stali*	Ni1	-	-	-	-
	Ni2	-	-	-	-
	Ni3	-	-	-	-
	Ni4	-	-	-	-
	Ni5	-	-	-	-
	A	1	-	1	100
	T	**1**		1	100
*Rhodnius* sp. 1*	Ni1	21	-	-	-
	Ni2	18	-	-	-
	Ni3	6	-	1	16,7
	Ni4	7	-	1	14,3
	Ni5	13	-	1	7,7
	A	-	-	-	-
	T	**65**		3	4,7
*Rhodnius* sp. 2*	Ni1	19	-	-	-
	Ni2	-	-	-	-
	Ni3	1	-	-	-
	Ni4	-	-	-	-
	Ni5	3	-	3	100
	A	-	-	-	-
	T	**23**		3	13
*Eratyrus mucronatus*	Ni1	-	-	-	-
	Ni2	-	-	-	-
	Ni3	1	-	-	-
	Ni4	-	-	-	-
	Ni5	-	-	-	-
	A	-	1	-	-
	T	**2**		-	-
**General total**		**116**		**16**	**13,8**

**Ni:** nymphs, **A:** adults, **T:** total triatomines collected by species, **N:** number of triatomines collected. * Species that have not reached the adult stage: (*Rhodnius* sp. 1, has a pattern of *R. pictipes* and *Rhodnius* sp. 2 has a pattern of *R. montenegrensis*).

Seven adult specimens were captured by 5 of the 43 family units, 3 of which were collected from the same household. Massaro et al.^5^left showcases with residents of a rural area of the municipality of Monte Negro, Rondônia, and obtained a sample of 11 specimens collected from 4 of 15 households.

As expected, triatomines were found in 100% of the studied palm trees because the palm tree selection was convenient. These data corroborate those of Meneguetti et al.^4^, who recorded 100% triatomine-positive palm trees in a study conducted in Rondônia, Southwest Amazon.

The present study recorded a mean density of 13.6 triatomines per palm tree, a number higher than that reported in a study performed in Monte Negro, Rondônia (3.4 triatomines per palm^5^) and lower than that reported in a study performed in Ouro Preto do Oeste, Rondônia (20.6 triatomines per palm)^4^.

Of the triatomine specimens collected in this study, 20.7% were *R. montenegrensis*; it was the most abundant species present in both palm trees and households. Its habitat is naturally associated with palm trees; however, it can also be found in households both in the rural^1^ and urban areas in Rio Branco, Acre^10^.

The species *R. stali* and *R. pictipes* each represented 0.9% of the triatomines collected in this study. *R. stali* was recently described in the city of Rio Branco, the state of Acre^11^. This species is one of the main vectors of Chagas disease in Bolivia (a country neighboring the state of Acre), with a large distribution throughout the country, and is associated with the transmission cycles that occur in local indigenous populations^11^, which can serve as a warning as there are several indigenous villages in the region of Cruzeiro do Sul, where the species has also been reported^12^. The species *R. pictipes* is a wild species with a wide distribution in South America^10^. A study conducted in Tocantins, in the Amazon biome, reported that this species is the most frequent in households (95.9% of the triatomines collected), and is more common in less anthropized places^13^, which can be characteristic of indigenous villages.

The wild species *E. mucronatus,* which was present in both palm trees and households, corresponded to 1.7% of the triatomine specimens collected in this study. Its domiciliation has already been demonstrated in the Apollo region, Bolivia, where it was infected by *T. cruzi,* particularly in the peridomicile^14^.

In the present study, 13.8% of the triatomines were infected with *T. cruzi*. Grouping the nymph stages 1, 2, and 3 of the 66 specimens, only 1 was infected (1.51%), while in the instar groups 4 and 5 and adults of the 50 specimens, 15 were infected (30%), showing a statistically significant difference (X^2^ = 19.41, p<0.001), indicating that the developmental stage of triatomines influences the percentage of *T. cruzi* infection*.* This outcome, owing to the high hematophagy performed during insect development until adulthood, increases the chances of trypanosomatid infections^4^ ^,^ ^15^. Similar observations have been made in a study by Meneguetti et al.^4^. There were no triatomines infected by *T. rangeli*.

The species with the highest percentage of infection, among all the species collected, were *R. stali* and *R. pictipes,* with each presenting 100% infection (however, it is to be noted that only one specimen of both species was collected), followed by *R. montenegrensis* (33.3%), which was the most abundant species collected in this study; its percentage of infection was 13.8% higher than all species together. Similar data were reported by Bilheiro et al.^15^, who showed a percentage of 30% in triatomines naturally infected by *T. cruzi* in *R. montenegrensis* specimens, captured in Monte Negro, Rondônia.

The presence of *E. mucronatus* and *R. montenegrensis* in both palm trees and households indicate the capacity of these species to exploit the human environment, mobility, and dispersal. As palm trees are important natural ecotypes for triatomines and environmental disturbance or destruction caused by humans, the household environment is susceptible to the presence of these insects, and consequently, they can play a significant role in the risk of human infection by *T. cruzi* ^4^.

Of the 11 triatomine species described in the state of Acre, 4 were present in the Boca do Moa community in Cruzeiro do Sul, a single community that is considered significant. This is because this community remains isolated for some months of the year owing to river flooding which reveals the potential of this area for future studies, and calls for a need for prophylaxis and measures to prevent vector and oral transmission of Chagas disease because this region contributes to most of the recorded cases in the Amazon region.
